# Anti-Human Herpesvirus 6 A/B Antibodies Titers Correlate With Multiple Sclerosis-Associated Retrovirus Envelope Expression

**DOI:** 10.3389/fimmu.2021.798003

**Published:** 2021-11-29

**Authors:** Silvia Pérez-Pérez, María I. Domínguez-Mozo, M. Ángel García-Martínez, M. Celeste García-Frontini, Noelia Villarrubia, Lucienne Costa-Frossard, Luisa M. Villar, Rafael Arroyo, Roberto Álvarez-Lafuente

**Affiliations:** ^1^ Environmental Factors in Degenerative Diseases Research Group, Hospital Clínico San Carlos, Instituto de Investigación Sanitaria del Hospital Clínico San Carlos (IdISSC), Madrid, Spain; ^2^ Immunology Department, Hospital Universitario Ramón y Cajal, Madrid, Spain; ^3^ Neurology Department, Hospital Universitario Ramón y Cajal, Madrid, Spain; ^4^ Neurology Department, Hospital Universitario Quironsalud Madrid, Madrid, Spain

**Keywords:** EBV, HERV-W, HHV-6A/B, multiple sclerosis, syncytin-1

## Abstract

Human endogenous retrovirus W family envelope proteins (pHERV-W ENV/syncytin-1) have been repeatedly associated with multiple sclerosis (MS). Here, we have focused on the study of pHERV-W ENV/syncytin-1 expression levels in MS patients (relapsing and progressive forms) and in healthy donors (HD) and on exploring their possible relationship with Epstein-Barr virus (EBV) and human herpesvirus-6A/B (HHV-6A/B). We included blood samples from 101 MS patients and 37 HD to analyze antiviral antibody titers by ELISA and pHERV-W ENV/syncytin-1 expression levels by flow cytometry as well as by qPCR. Patients with relapsing MS forms showed significantly higher pHERV-W ENV/syncytin-1 protein and gene expression levels than HD. Progressive MS patients also showed significantly higher protein and gene expression levels than both HD and relapsing MS patients. Regarding antiviral antibodies titers, anti-HHV-6A/B IgM levels were positively correlated with pHERV-W ENV/syncytin-1 protein expression levels in patients with relapsing MS, while in the progressive forms patients this correlation was found with anti-HHVA/B IgG levels. Therefore, pHERV-W ENV could be involved in MS pathogenesis, playing a role in relapsing and progressive forms. Besides, anti-HHV-6A/B antibodies positively correlated with pHERV-W ENV expression. Further studies are needed to better understand this possible relationship.

## 1 Introduction

Multiple sclerosis (MS) is a chronic, autoimmune and demyelinating disease that affects the central nervous system (CNS). It is mainly characterized by lesions in the myelin of neuronal axons, threatening their integrity and causing irreversible damage to them. In spite of its huge variability, MS manifests mainly with episodes of neurological dysfunction, called relapses, followed by partial or complete recovery (relapsing-remitting MS, RR-MS); however, in certain cases, there could exist a progressive degeneration from the onset of MS (primary progressive MS, PP-MS) (1).

Despite being discovered more than a century ago, MS etiopathogenesis is partially unknown. At present, certain environmental factors are considered to develop an abnormal immune response in genetically susceptible individuals (2). One of the most studied environmental factors are viruses, and especially herpesviruses – such as the Epstein-Barr virus (EBV) and the human herpesvirus 6A/B (HHV-6A/B) – or human endogenous retroviruses (HERVs).

There are a considerable number of studies supporting the involvement of both EBV and HHV-6A/B in MS. Nevertheless, the actual mechanisms by which they may act are not completely understood (3). One of the theories involves the possible transactivation of HERVs (4).

HERVs come from exogenous retroviruses that integrated their genome into human germinal lines thousands of years ago. Currently, they constitute around 8% of the human genome (5). The HERV-W family, and its two main members (multiple sclerosis-associated retrovirus, MSRV, and ERVWE1), have been the most studied ones concerning MS. While MSRV (whose genomic location is unknown) is able to form complete viral particles, ERVWE1 (located at 7q21.2) has inactivating mutations that prevent their formation. Moreover, ERVWE1 envelope protein (env), called syncytin-1, is a fusogenic protein essential for placental syncytiotrophoblast formation (6). Since MSRV env location is unknown, all of the HERV-W env sequences except for syncytin-1 are referred to as pHERV-W ENV. pHERV-W ENV and syncytin-1 show a high degree of homology (94%), so it is impossible to differentiate them at the protein level with nowadays commercial antibodies (7). In relation to MS, proinflammatory and neurotoxic properties of pHERV-W ENV could lead to the development and progression of the disease (8).

Therefore, our aim in this study was to compare the levels of protein and gene expression of pHERV-W ENV/syncytin-1 in RR-MS, PP-MS, and healthy donors (HD), and to analyze their possible relationship with anti-EBV and anti-HHV-6A/B antibody titers. We concluded that pHERV-W ENV could be involved in MS pathogenesis, playing a role both in relapsing and progressive forms. Besides, the positive correlation found between HHV-6A/B and pHERV-W ENV expression could support a possible transactivation process.

## 2 Materials and Methods

### 2.1 Patients and Samples

We performed a cross-sectional study including 80 RR-MS patients, 21 PP-MS patients, and 37 HD diagnosed at “Hospital Clínico San Carlos” (Madrid, Spain) and “Hospital Universitario Ramón y Cajal” (Madrid, Spain) according to updated McDonald criteria (9). **Table 1** shows the demographic and clinical characteristics of the included individuals. The study was conducted according to the guidelines of the Declaration of Helsinki, and approved by our local Ethic Committee: Comité Ético de Investigación Clínica del Hospital Clínico San Carlos (C.P. - C.I. 16/070-E). Informed consents were obtained from all subjects involved in the study.

**Table 1:** Demographic and clinical characteristics of patients and HD included in this study.

	RR-MS	PP-MS	HD
n	80	21	37
Sex (% females)	62.5	61.9	56.8
Age (y.o., mean ± SD)	40.3 ± 8.3	55.4 ± 11.0	40.4 ± 7.0
Disease duration [m, median (P25 - P75)]	102.5 (53.0 -166.0)	84.0 (30.0 – 156.0)	–
Treatment (%):			
First line (interferon, glatiramer acetate)	60.0	9.5	–
Second line (natalizumab)	35.0	–	–
Without treatment	5.0	90.5	–
Treatment duration [m, median (P25 - P75)]:			
First line	34.0 (19.3 – 74.5)	20.0 *	–
Second line	17.0 (8.5 – 23.8)	–	–
EDSS [median (P25 - P75)]	2.0 (1.0 – 3.9)	3.2 (2.5 – 6.0)	–
MSSS [median (P25 - P75)]	2.1 (0.7 – 4.6)	5.7 (3.9 – 7.9)	–
Annualized relapse rate [median (P25 - P75)]	0.6 (0.3 – 0.9)	–	–

y.o., years old; m: months; P, percentile; SD, standard deviation.

*Too small sample size to determine P25 y P75.

We collected peripheral blood samples from each enrolled patient. For serum obtention, one dry tube was collected, centrifuged (900 g, 15 min), and stored at -80°C. In addition, one cell preparation tube (CPT™, BD Vacutainer) was collected for peripheral blood mononuclear cells (PBMCs) isolation using density gradient centrifugation (920 g, 30 min); afterward, PBMCs were cryopreserved in fetal bovine serum (FBS) with dimethyl sulfoxide (DMSO) (10%) and stored in liquid nitrogen (-176°C).

### 2.2 Variables of the Study

#### 2.2.1 Laboratory Variables

pHERV-W ENV/syncytin-1 protein expression, *pHERV-W ENV* and *syncytin-1* gene expression, and titers of anti-VCA (EBV viral capsid antigen) IgG, anti-EBNA-1 (Epstein-Barr nuclear antigen) IgG, anti-HHV-6A/B IgM, and anti-HHV-6A/B IgG antibodies.

#### 2.2.2 Demographic Variables

Sex and age.

#### 2.2.3 Clinical Variables

MS presence/absence, MS clinical form, age of onset of MS, disease duration, treatment, disability (according to EDSS – expanded disability status scale – and MSSS – multiple sclerosis severity score), and presence/absence of relapses.

### 2.3 Enzyme-Linked Immunosorbent Assay (ELISA)

We used commercial kits for the detection of IgG against VCA and EBNA-1 of EBV (*Captia*™; *Trinity Biotech*, Wicklow, Ireland), as well as for the detection of IgG and IgM against HHV-6A/B (*Vidia, Ltd*.; Czech Republic), following in both cases the manufacturer’s instructions, including the use of negative controls and standards.

### 2.4 Flow Cytometry

For flow cytometry experiments, 500,000 PBMCs were used to determine pHERV-W ENV/syncytin-1 protein expression through an indirect staining method. Firstly, Fc receptors were blocked using 2.5 μl of *Human TruStain FcX* (BioLegend Cat# 422302, RRID : AB_2818986) (10 min, RT). Afterwards, 3 μl of primary monoclonal antibody anti-pHERV-W ENV/syncytin-1 (Sigma-Aldrich Cat# WH0030816M6, RRID : AB_1841512) were added (30 min, 4°C). After washing, a secondary PE-labelled antibody against murine IgG (BioLegend Cat# 406608, RRID : AB_10551618) was added (20 min, 4°C). PBMCs were washed again and the secondary antibody-free places were blocked with 20 μl of murine IgG (Sigma-Aldrich Cat# I5381, RRID : AB_1163670) (15 min, RT). Finally, PBMC were stained with a set of monoclonal antibodies against the following surface markers: CD3-PerCP (BD Biosciences Cat# 345766, RRID : AB_2783791), CD14-FITC (BD Biosciences Cat# 345784, RRID : AB_2868810), CD19-APC (BD Biosciences Cat# 345791, RRID : AB_2868817), CD45-APC-H7 (BD Biosciences Cat# 641417, RRID : AB_2800453) and CD56-BV421™ (BD Biosciences Cat# 562751, RRID : AB_273205).

Stained PBMC were analyzed in a *Gallios* flow cytometer and data analysis was performed using *Kaluza Flow Cytometry Analysis Software* (GalliosTM Kaluza, RRID : SCR_016700), both supplied by *Becton Dickinson* (Franklin Lakes, NJ, USA). An average of 50,000 events per sample was analyzed. For gating, a first selection of monocytes and lymphocytes was made to exclude debris and apoptotic/dead cells, followed by the selection of singlets and CD45+ (PBMCs marker) cells.

pHERV-W ENV/syncytin-1 protein expression levels were analyzed in each of the following cell populations: monocytes, B lymphocytes, T lymphocytes, and NK cells.

### 2.5 qPCR for *pHERV-W ENV* and *Syncytin-1* Gene Expression Quantification

Firstly, RNA was isolated from PBMCs using the *QIAamp RNA Blood Mini* kit (*Qiagen*, Valencia, CA, USA) and treated with the *TURBO DNA-free*™ (*Invitrogen*, Carlsbad, CA, USA), following in both cases the manufacturer’s instructions. Afterward, cDNA was obtained using the *Transcriptor First Strand cDNA Synthesis kit* (*Roche Diagnostics*, S.L., Barcelona, Spain), where negative controls (without retrotranscriptase) were included.

Quantitative real-time polymerase chain reactions (qPCR) were performed using the *TaqMan*™ *Gene Expression Master Mix* system (*Thermo Fisher Scientific*, Waltham, MA, USA) in a *Rotor-Gene3000* (*Corbett Research*, Sydney, Australia). We used previously published primers, probes, and conditions for *pHERV-W ENV* and *syncytin-1* detection (7). Glucuronidase B was used as the housekeeping gene (*Human GUSB Endogenous Control*; *Thermo Fisher Scientific*, Waltham, MA, USA). For relative quantification, the 2^-ΔΔCt^ (10) method was applied. Finally, negative controls were included in each assay, and samples with cycle threshold values above 40 were considered negative.

### 2.6 Statistics

Continuous variables were expressed as mean ± SD (standard deviation) – if they were parametric – or as median (25th, 75th percentile) – if they were not –, while categorical variables were expressed as percentages.

We analyzed pHERV-W ENV/syncytin-1 protein and gene expression differences among RR-MS patients, PP-MS patients, and HD. For parametric continuous variables, the t-Student’s test was used; otherwise, we used the Mann-Whitney U test.

Moreover, we studied the possible link between antiviral antibodies titers and pHERV-W ENV/syncytin-1 expression in RR-MS patients, PP-MS patients, and HD; for antibodies titers, only positive values were used in the analyses, except for anti-HHV-6A/B IgM titers, due to low seroprevalence reasons. We also analyzed the possible impact of demographic, clinical, and radiological features on these relationships, stratifying populations accordingly; for continuous variables, the median values were selected as the cut-offs, except for age (the cut-off was 45 years old, proposed as the possible immunosenescence onset for MS patients) and EDSS (the cut-off was 3, proposed as the onset of irreversible disability). We used the Pearson correlation coefficient for combinations of two parametric variables and the Spearman’s rank correlation coefficient for the non-parametric ones.

Demographic, clinical, and radiological variables were also taken into account for their possible effect as confounding factors by conducting linear regression models when cohorts were not homogeneous for those variables.

Subjects with missing data were omitted from the corresponding analyses. P-values < 0.05 were considered as statistically significant. All analyses were performed using SPSS version 21.0 (IBM SPSS Statistics, RRID : SCR_019096) and graphs were made using Prism version 5.0 (GraphPad Prism, RRID : SCR_002798).

## 3 Results

### 3.1 pHERV-W ENV/Syncytin-1 Protein and Gene Expression in RR-MS Patients, PP-MS Patients, and HD

**Figure 1:**
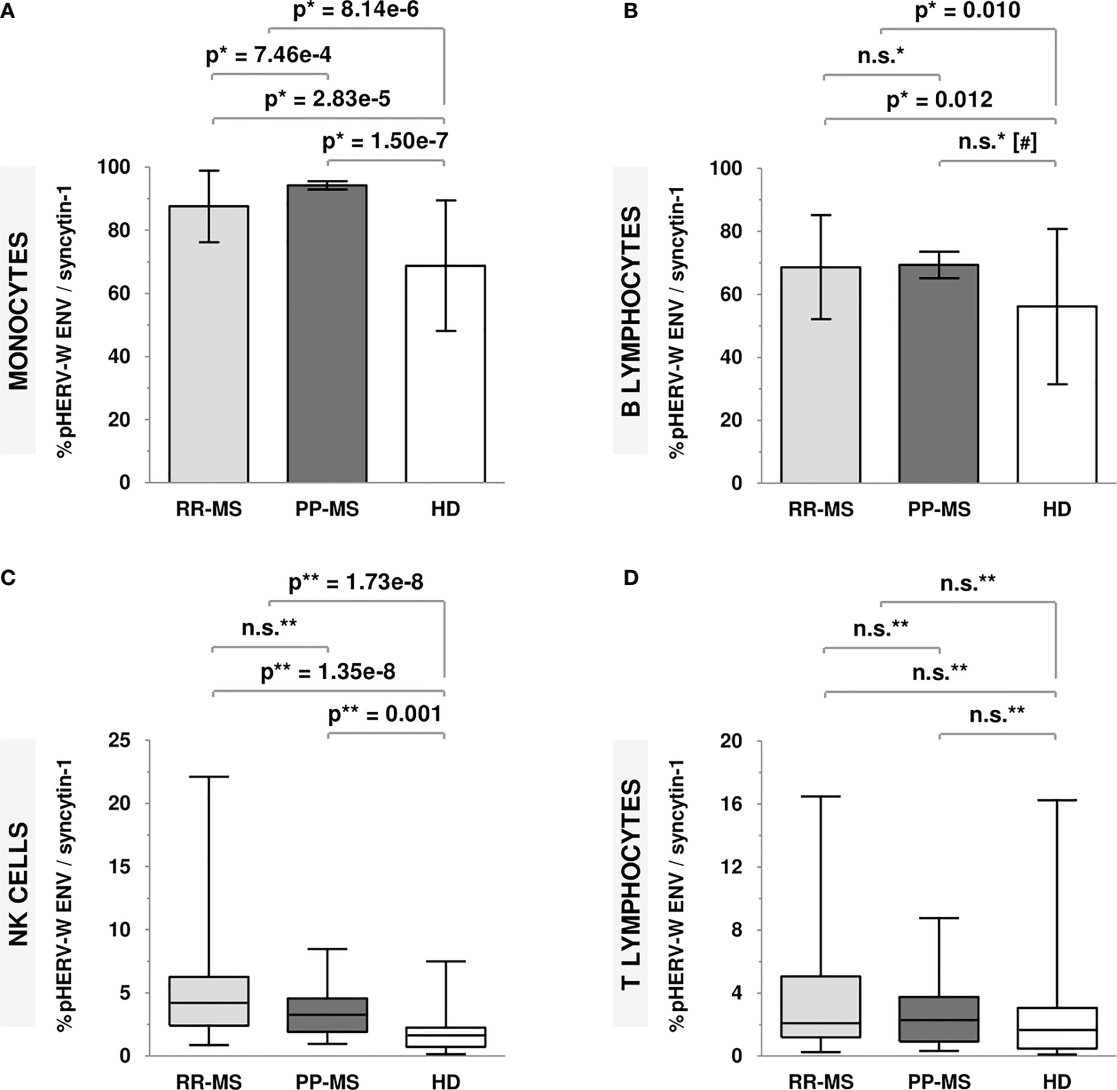
Protein expression levels of pHERV-W ENV/syncytin-1 in RR-MS patients, PP-MS patients and HD. **(A)** Monocytes (mean ± SD): RR-MS = 87.54 ± 11.36; PP-MS= 94.24 ± 5.97; HD = 68.76 ± 20.70. **(B)** B lymphocytes (mean ± SD): RR-MS = 68.64 ± 16.53; PP-MS = 69.32 ± 18.98; HD = 56.12 ± 24.65. **(C)** NK cells [median (P25-P75)]: RR-MS = 4.19 (2.40-6.25); PP-MS = 3.26 (1.92-4.55); HD = 1.63 (0.74-2.24). **(D)** T lymphocytes [median (P25-P75)]: RR-MS = 2.08 (1.19-5.07); PP-MS = 2.29 (0.92-3.75); HD = 1.67 (0.48-3.06). [*t-Student test; **Mann-Whitney U test; (#) age-adjusted p-value; n.s., not significant].

pHERV-W ENV/syncytin-1 protein expression levels were significantly higher in MS patients compared to HD. As it is shown in **Figure 1**, this increase was found in every analyzed cell population (monocytes, p=8.14e-6; B lymphocytes, p=0.010; NK cells, p=1.73e-8) except for T lymphocytes.

Considering only RR-MS patients (**Figure 1**), results were almost the same as those previously described, with significantly higher pHERV-W ENV/syncytin-1 protein expression levels in RR-MS patients compared to HD (monocytes, p=2.83e-5; B lymphocytes, p =0.012; NK cells, p=1.35e-8).

Relating to PP-MS patients (**Figure 1**), they showed significantly higher protein expression levels in monocytes (p=1.50e-7) and NK cells (p=0.001) compared to HD. Moreover, these patients showed significantly higher pHERV-W ENV/syncytin-1 protein expression levels in monocytes (p=7.46e-4) compared to RR-MS patients.

**Figure 2:**
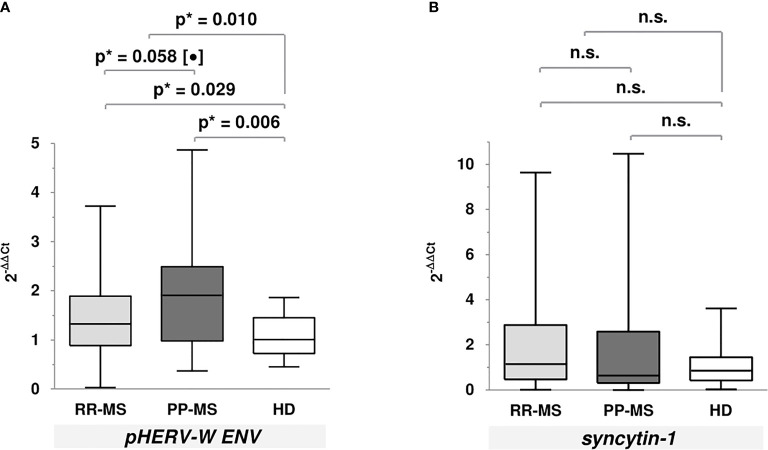
Gene expression levels of *pHERV-W ENV* and *syncytin-1* using the 2^-ΔΔCt^ method. **(A)** *pHERV-W ENV* [median (P25-P75)]: RR-MS = 1.33 (0.89-1.89); PP-MS = 1.91 (0.98-2.49); HD = 1.01 (0.72-1.45). **(B)** *Syncytin-1* [median (P25-P75)]: RR-MS = 1.15 (0.47-2.88); PP-MS = 0.64 (0.31-2.59); HD =0.86 (0.42-1.45). [*Mann-Whitney U test; (•) tendency to signification; n.s., not significant].

Regarding gene expression levels (**Figure 2**), we only found that MS patients (p=0.010), both RR-MS (p=0.029) and PP-MS (p=0.006), showed significantly higher *pHERV-W ENV* gene expression levels compared to HD; moreover, levels were higher in PP-MS patients compared to the RR-MS ones, showing a trend to statistical significance. We found no significant differences in *syncytin-1* gene expression levels.

### 3.2 Relation Between pHERV-W ENV/Syncytin-1 Protein/Gene Expression and Titers of Antibodies Against EBV and HHV-6A/B in RR-MS, PP-MS, and HD

**Figure 3:**
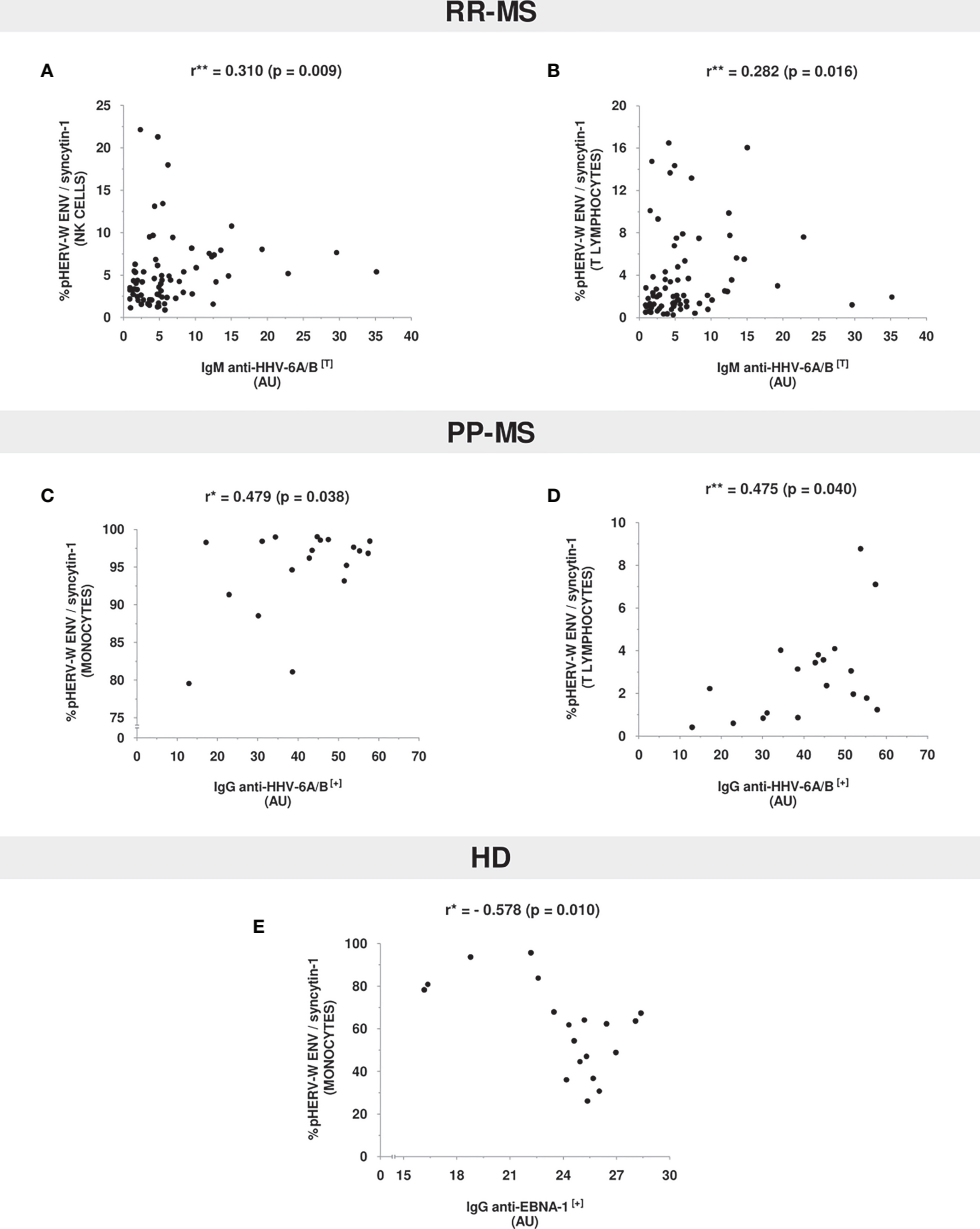
Correlation plots between antiviral antibodies titers and pHERV-W ENV/syncytin-1 protein expression levels in **(A, B)** RR-MS patients, **(C, D)** PP-MS patients, and **(E)** HD. Only statistically significant correlations are shown. [*Pearson’s Correlation Coefficient; **Spearman’s Correlation Coefficient; (+), positive values (≥11 AU); (T), total values (positive&negative); AU, arbitrary units].

Regarding RR-MS patients (**Figures 3A, B**), we found a positive significant correlation between anti-HHV-6A/B IgM antibodies titers and pHERV-W ENV/syncytin-1 protein expression levels in NK cells (r=0.310, p=0.009) and T lymhpocytes (r=0.282, p=0.016).

On the other hand, PP-MS patients (**Figures 3C, D**) showed a positive significant correlation between anti-HHV-6A/B IgG antibodies titers and pHERV-W ENV/syncytin-1 protein expression levels in monocytes (r=0.479, p=0.038) and T lymphocytes (r=0.475, p=0.040).

Relating to HD (**Figure 3E**), and contrary to the aforementioned trends, there was a negative significant correlation between anti-EBNA-1 IgG antibodies titers and pHERV-W ENV/syncytin-1 protein expression levels in monocytes (r=-0.578, p=0.010).

Finally, due to the small sample size of the PP-MS patients and the HD cohorts, we could only analyze the possible impact of demographic, clinical, and radiological factors on the aforementioned correlations in the RR-MS cohort. In those patients, correlations seemed to be more associated with the following factors: age < 45 years old, age of MS onset < median (30 years old), duration of the disease < median (102 months), EDSS ≥ 3, presence of relapses in the two previous years and absence of gadolinium-enhancing lesions. Data can be seen in **Supplementary Figure 1**.

## 4 Discussion

Despite MS etiopathogenesis is still partially unknown, it is undeniable that there exists an autoimmune process in the CNS which leads to the destruction of oligodendrocytes and myelin sheaths. In addition, it is accepted that both environmental factors and genetics interplay in MS development. Researchers have repeatedly described the potential role of viruses (especially herpesviruses) in MS. For example, it has been shown how EBV can trigger a specific humoral immune response in MS patients, more intensely than in HD or in other neurological diseases patients; furthermore, anti-EBNA-1 IgG antibodies titers seem to be increased in MS patients compared to HD, decreasing after treatment with interferon (11, 12). These antibodies are also negatively correlated to B lymphocyte activating factor (BAFF) levels, which appear to be associated with more stable disease (13). However, the actual mechanisms of action of viruses in MS are unknown and many theories have been proposed in this regard (14), such as their role in overstimulating immune responses (15) or a possible cross-reaction with myelin proteins due to molecular mimicry (16). One of the theories proposed involves their possible role as transactivators of HERV elements, such as the HERV-W family envelope proteins.

Since their identification, many studies have pointed out a possible link between pHERV−W ENV/syncytin-1 and MS (17). In fact, antibodies against pHERV-W ENV have even been proposed as biomarkers to discriminate MS from other diseases, especially neuromyelitis optica, since titers are significantly higher in MS patients (18, 19).

In line with previously published investigations (20, 21), here we have observed significantly higher pHERV−W ENV/syncytin-1 protein expression levels in PBMC from MS patients compared to HD. This rise has been observed in every studied population except for T lymphocytes, which barely expressed these proteins.

PP-MS patients have shown significantly higher pHERV-W ENV/syncytin-1 protein expression levels in monocytes compared to RR-MS too. Previous studies have linked MSRV presence in MS patients to a worse prognosis and greater progression and severity of the disease (22, 23); besides, the phase 2b clinical assay carried out with temelimab (GNbAC1, anti-pHERV-W ENV monoclonal antibody) supports the use of this treatment in progressive MS forms because of its potential anti-neurodegenerative effect (24). Although monocytes have not been especially linked to PP-MS in the literature, proteins such as sialoadhesin-1 have been reported to be increased in those patients compared to the RR-MS ones (25). In addition, this overexpression in monocytes could lead to a higher antigen presentation contributing to promoting the immune response. However, it is an interesting result to delve into in future studies.

Relating to gene expression results, both RR-MS and PP-MS patients showed significantly increased *pHERV-W ENV* gene expression levels compared to HD, as well as PP-MS patients compared to the RR-MS ones. However, no differences were found in terms of *syncytin-1* gene expression. These results are in line with previous studies (7) and they reinforce the possible link between pHERV-W ENV and MS development. Taking these results into account, we may hypothesize that the pHERV-W ENV/syncytin-1 protein increase could be a result of a rise in *pHERV-W ENV* gene expression. However, the lack of specific commercial available antibodies prevents us from validating this theory.

In spite of their sequence homology and sharing many properties and features, they show some functional and structural differences to take into consideration. For example, while the transmembrane domine of syncytin-1 has fusogenic properties, it is not functional in pHERV-W ENV. On the other hand, while syncytin-1 is a membrane protein, pHERV-W ENV can be both membrane-associated and soluble (forming hexamers, trimers, or monomers) (26, 27). Therefore, the same as only syncytin-1 is involved in the syncytiotrophoblast formation, only pHERV-W ENV proteins would take a part in MS pathogeny. However, other investigators disagree and propose a possible involvement of syncytin-1 in MS (28).

The possible involvement of pHERV-W ENV proteins in MS could be justified by their proinflammatory properties. These proteins, through TLR4 receptors, could promote adhesion, diapedesis, and infiltration of immune cells into the CNS, activate microglia cells, induce cytokine production, or damage oligodendrocyte precursors, preventing remyelination (29). Moreover, this overexpression could contribute, for example, to an increase in the antigen-presentation rate in monocytes or to the aberrant activation of cells which barely express pHERV-W ENV proteins, such as T lymphocytes, through proinflammatory factors release (in fact, CD4+ T lymphocyte activation through TLR4 receptors leads to a boost of autoimmune processes in experimental autoimmune encephalomyelitis, the mouse model of MS (30)). In general, these proteins would promote a proinflammatory environment and an aberrant and sustained immune response.

Another theory that would support the involvement of pHERV-W ENV in MS could be based on a cross-reaction between these proteins and MOG (myelin oligodendrocyte glycoprotein). *In silico* studies have described that these proteins show a huge sequence homology and that they perfectly fit in the antigen presentation pocket of HLA class II molecules (31, 32). Therefore, antibodies against pHERV-W ENV could also attack MOG, as there has been seen in other *in vivo* studies (33). In contrast, in HD there would be a generalized immunotolerance towards pHERV-W ENV proteins, avoiding the immune response against these proteins (8).

Although in the literature it is commonly found that there could be a link between pHERV-W ENV and MS, there are also some opposed opinions. Some studies have not found significant differences between MS patients and HD in terms of pHERV-W ENV expression levels (34–36). Thus, there is not a complete consistency among the studies up to date, which justifies continuing delving into this matter.

As regards the possible relationship between antiviral antibodies and pHERV-W ENV/syncytin-1 expression, we have found a positive correlation between anti-HHV-6A/B antibodies titers and pHERV-W ENV/syncytin-1 protein expression levels in MS patients. Furthermore, this relationship was found with anti-HHV-6A/B IgM antibodies in RR-MS patients and with anti-HHV-6A/B IgG antibodies in PP-MS patients.

In RR-MS patients, a moderate positive correlation was found between anti-HHV-6A/B IgM antibodies and pHERV-W ENV protein expression levels in NK cells and T lymphocytes. The relationship between HHV-6A/B and NK cells has been profoundly studied by Dr. Di Luca’s group. They have shown how HHV-6A/B is able to regulate the expression of several miRNAs related to autoimmune and inflammatory processes (37, 38). As far as T lymphocytes are concerned, it is the most suitable cell type for HHV-6A/B replication, so this would be an expectable result.

On the other hand, PP-MS patients showed a moderate-strong positive correlation between anti-HHV-6A/B IgG antibodies titers and pHERV-W ENV/syncytin-1 expression in T lymphocytes and monocytes. Monocytes are one of the main reservoirs for HHV-6A/B. However, taking into account that monocytes are not particularly related to PP-MS, once again it is an interesting result to delve into in further studies.

On the contrary, in the HD cohort, we found a negative correlation between anti-EBNA-1 antibodies and pHERV-W ENV/syncytin-1 expression levels in monocytes. Despite being a result to be confirmed in further studies, this fact could involve a possible opposite role of the analyzed herpesviruses in MS patients and HD.

Due to the small sample size of the PP-MS and the HD cohorts, we could only analyze the impact of demographic and clinical factors in the RR-MS cohort. In light of the results, previously found correlations seemed to be more related to the following factors: age under 45 years old (both males and females), early age of disease onset (under the median of the RR-MS cohort, 30 years old), short disease evolution (under the median of the complete cohort, 102 months), EDSS ≥ 3, presence of relapses during the two previous years and absence of gadolinium-enhancing lesions. Having found clearer correlations in those patients with an earlier disease debut, an EDSS ≥ 3 or who recently suffered relapses might be related to the role of the relationship between HHV-6A/B infection and pHERV-W ENV expression in an active MS. In general, we have found a consistent, direct relationship between anti-HHV-6A/B antibodies titers and the expression of pHERV-W ENV, therefore it is remarkable to have repeatedly found this link throughout the aforementioned analyses, especially in both NK cells and T lymphocytes. These results could support a possible role of HHV-6A/B upon pHERV-W ENV transactivation in these cell populations.

Other groups have described a possible role of HHV-6A/B upon pHERV-W ENV expression transactivation. Charvet et al. (39) have proposed that HHV-6A, through CD46 receptor, could trigger a signaling pathway that would promote pHERV-W ENV expression and, finally, would lead to tissular damage. However, other investigators have found no link between HHV-6A/B and pHERV-W ENV (40).

Regarding CNS, viruses might penetrate inside immune cells like a Trojan horse. There, these virus-carrier immune cells would develop an inflammatory environment where proinflammatory factors would induce pHERV-W ENV sequences expression. This overexpression would lead to even more proinflammatory factors release, creating a neuroinflammatory loop that would provoke structural damage (41). While in a healthy CNS these reactions would be under control, in MS patients the altered immune system would promote an aberrant and exacerbated immune reaction (42).

Concerning the limitations of this study, although we have tried to minimize the possible confounding agents effects, HERV elements expression is characterized by a huge variability. There exists a high genetic-based interindividual variability in HERV expression (35). Furthermore, diverse factors have been described to promote or inhibit HERV expression (microbiota microorganisms; chemical agents, such as aspirin or caffeine; biological modulation, such as epigenetics; etc.) (43–45).

Another factor to be considered is the small sample size of the HD cohort compared to that of the RR-MS. During this project, the commercial antibody used for pHERV-W ENV/syncytin-1 analyses was discontinued. This fact forced us to terminate the analyses of HD samples and, therefore, this cohort is not as large as we had originally planned. Besides, the limited radiological data that we could gather during the collection of information from medical records should also be taken into account. Therefore, this small sample size may be the reason for not having found significant relationships in patients with gadolinium-enhancing lesions, as we have found in some previous studies (46).

Regarding MS patient treatment, it would be useful to assess the possible effect of the different therapies on the aforementioned results. It has been published that long-term treatment with natalizumab is able to reduce the humoral immune response to pHERV-W ENV antigens (47), as well as interferon therapy can reduce this response after six months of treatment (48). The median duration of treatment with natalizumab in our patients was 17 months, while for interferon it was 36 months. By conducting a longitudinal study, we would be able to evaluate the potential effect of these treatments on pHERV-W ENV/syncytin-1 levels and their correlation with antiviral antibodies.

Although in this study we have analyzed antibody titers against HHV-6A/B together, using available ELISA commercial kits, a recently published paper has analyzed the titers against HHV-6A and HHV-6B separately, using a novel multiplex serological assay. Thus, it would be of great interest to determine in future studies whether HHV-6A or HHV-6B could correlate with pHERV-W ENV/syncytin-1 expression (49).

Despite we have studied several variables and performed the corresponding tests, we have not applied corrections for multiple comparisons due to the exploratory nature of this study.

All in all, in light of the results, pHERV-W ENV could be involved in MS pathogenesis, playing a role in relapsing and progressive forms. Besides, the positive correlation found between HHV-6A/B and pHERV-W ENV expression could support previous findings of a possible transactivation process. Moreover, it should be pointed out that this work is one of the most detailed studies performed up to date relating to the possible involvement of pHERV-W ENV in MS, as well as its possible link to HHV-6A/B, using MS samples, both from RR-MS and PP-MS patients, and HD.

## Data Availability Statement

The raw data supporting the conclusions of this article will be made available by the authors, without undue reservation.

## Ethics Statement

The studies involving human participants were reviewed and approved by Comité Ético de Investigación Clínica del Hospital Clínico San Carlos (C.P. - C.I. 16/070-E). The patients/participants provided their written informed consent to participate in this study.

## Author Contributions

Conceptualization, SP-P and RA-L. Methodology, SP-P, MID-M, MAG-M, MG-F, and N.V. Software, SP-P and MID-M. Validation, SP-P and MID-M. Formal analysis, SP-P and MID-M. investigation, SP-P, MID-M, MAG-M, and MG-F. Resources, NV, LC-F, LMV, RA, and RA-L. Data curation, SP-P, and MID-M. Writing-original draft preparation, SP-P. Writing-review and editing, MID-M and RA-L. Visualization, SP-P. Supervision, LMV, RA, and RA-L. Project administration, LMV, RA, and RA-L. Funding acquisition, LC-F, LMV, RA, and RA-L All authors contributed to the article and approved the submitted version.

## Funding

This work was financially supported by Instituto de Salud Carlos III (ISCIII)-Fondo Europeo de Desarrollo Regional (Feder) (PI18/00204), “REEM: Red Española de Esclerosis Múltiple” (RD16/0015/0013), “Fundación Ramón Areces” (CIVP18A3860), and “Fundación LAIR”.

## Conflict of Interest

The authors declare that the research was conducted in the absence of any commercial or financial relationships that could be construed as a potential conflict of interest.

## Publisher’s Note

All claims expressed in this article are solely those of the authors and do not necessarily represent those of their affiliated organizations, or those of the publisher, the editors and the reviewers. Any product that may be evaluated in this article, or claim that may be made by its manufacturer, is not guaranteed or endorsed by the publisher.

## References

[1] Thompson AJ Baranzini SE Geurts J Hemmer B Ciccarelli O. Multiple Sclerosis. Lancet (2018) 391:1622–36. doi: 10.1016/S0140-6736(18)30481-1 29576504

[2] Dobson R Giovannoni G. Multiple Sclerosis - a Review. Eur J Neurol (2019) 26:27–40. doi: 10.1111/ene.13819 30300457

[3] Tarlinton RE Martynova E Rizvanov AA Khaiboullina S Verma S. Role of Viruses in the Pathogenesis of Multiple Sclerosis. Viruses (2020) 12. doi: 10.3390/v12060643 PMC7354629 32545816

[4] Jakhmola S Upadhyay A Jain K Mishra A Jha HC. Herpesviruses and the Hidden Links to Multiple Sclerosis Neuropathology. J Neuroimmunol (2021) 358:577636. doi: 10.1016/j.jneuroim.2021.577636 34174587

[5] Christensen T. Human Endogenous Retroviruses in Neurologic Disease. APMIS Acta Pathol Microbiol Immunol Scand (2016) 124:116–26. doi: 10.1111/apm.12486 26818266

[6] Antony JM Deslauriers AM Bhat RK Ellestad KK Power C. Human Endogenous Retroviruses and Multiple Sclerosis: Innocent Bystanders or Disease Determinants? Biochim Biophys Acta (2011) 1812:162–76. doi: 10.1016/j.bbadis.2010.07.016 PMC7172332 20696240

[7] Mameli G Poddighe L Astone V Delogu G Arru G Sotgiu S. Novel Reliable Real-Time PCR for Differential Detection of MSRVenv and Syncytin-1 in RNA and DNA From Patients With Multiple Sclerosis. J Virol Methods (2009) 161:98–106. doi: 10.1016/j.jviromet.2009.05.024 19505508

[8] Dolei A Perron H. The Multiple Sclerosis-Associated Retrovirus and its HERV-W Endogenous Family: A Biological Interface Between Virology, Genetics, and Immunology in Human Physiology and Disease. J Neurovirol (2009) 15:4–13. doi: 10.1080/13550280802448451 19039700

[9] Polman CH Reingold SC Banwell B Clanet M Cohen JA Filippi M. Diagnostic Criteria for Multiple Sclerosis: 2010 Revisions to the McDonald Criteria. Ann Neurol (2011) 69:292–302. doi: 10.1002/ana.22366 21387374 PMC3084507

[10] Schmittgen TD Livak KJ. Analyzing Real-Time PCR Data by the Comparative. CT method Nat Protoc (2008) 3:1101–8. doi: 10.1038/nprot.2008.73 18546601

[11] Mameli G Cocco E Frau J Marrosu MG Sechi LA. Epstein Barr Virus and Mycobacterium Avium Subsp. Paratuberculosis Peptides are Recognized in Sera and Cerebrospinal Fluid of MS Patients. Sci Rep (2016) 6:22401. doi: 10.1038/srep22401 26956729 PMC4783662

[12] Mameli G Cossu D Cocco E Masala S Frau J Marrosu MG. EBNA-1 IgG Titers in Sardinian Multiple Sclerosis Patients and Controls. J Neuroimmunol (2013) 264:120–2. doi: 10.1016/j.jneuroim.2013.07.017 24099984

[13] Mameli G Cocco E Frau J Arru G Caggiu E Marrosu MG. Serum BAFF Levels, Methypredsinolone Therapy, Epstein-Barr Virus and Mycobacterium Avium Subsp. Paratuberculosis Infection in Multiple Sclerosis Patients. Sci Rep (2016) 6:29268. doi: 10.1038/srep29268 27383531 PMC4935889

[14] Libbey JE Cusick MF Fujinami RS. Role of Pathogens in Multiple Sclerosis. Int Rev Immunol (2014) 33:266–83. doi: 10.3109/08830185.2013.823422 PMC4369909 24266364

[15] Cossu D Mameli G Galleri G Cocco E Masala S Frau J. Human Interferon Regulatory Factor 5 Homologous Epitopes of Epstein-Barr Virus and Mycobacterium Avium Subsp. Paratuberculosis Induce a Specific Humoral and Cellular Immune Response in Multiple Sclerosis Patients. Mult Scler J (2015) 21:984–95. doi: 10.1177/1352458514557304 25392335

[16] Mameli G Cossu D Cocco E Masala S Frau J Marrosu MG. Epstein-Barr Virus and Mycobacterium Avium Subsp. Paratuberculosis Peptides are Cross Recognized by Anti-Myelin Basic Protein Antibodies in Multiple Sclerosis Patients. J Neuroimmunol (2014) 270:51–5. doi: 10.1016/j.jneuroim.2014.02.013 24642384

[17] Perron H Germi R Bernard C Garcia-Montojo M Deluen C Farinelli L. Human Endogenous Retrovirus Type W Envelope Expression in Blood and Brain Cells Provides New Insights Into Multiple Sclerosis Disease. Mult Scler Houndmills Basingstoke Engl (2012) 18:1721. doi: 10.1177/1352458512441381 PMC3573672 22457345

[18] Arru G Sechi E Mariotto S Zarbo IR Ferrari S Gajofatto A. Antibody Response Against HERV-W in Patients With MOG-IgG Associated Disorders, Multiple Sclerosis and NMOSD. J Neuroimmunol (2020) 338:577110. doi: 10.1016/j.jneuroim.2019.577110 31715457

[19] Arru G Sechi E Mariotto S Farinazzo A Mancinelli C Alberti D. Antibody Response Against HERV-W Env Surface Peptides Differentiates Multiple Sclerosis and Neuromyelitis Optica Spectrum Disorder. Mult Scler J - Exp Transl Clin (2017) 3:2055217317742425. doi: 10.1177/2055217317742425 29204291 PMC5703109

[20] Garcia-Montojo M Rodriguez-Martin E Ramos-Mozo P Ortega-Madueño I Dominguez-Mozo MI Arias-Leal A. Syncytin-1/HERV-W Envelope Is an Early Activation Marker of Leukocytes and is Upregulated in Multiple Sclerosis Patients. Eur J Immunol (2020) 50(5):685–94. doi: 10.1002/eji.201948423 32012247

[21] Brudek T Christensen T Aagaard L Petersen T Hansen HJ Møller-Larsen A. B Cells and Monocytes From Patients With Active Multiple Sclerosis Exhibit Increased Surface Expression of Both HERV-H Env and HERV-W Env, Accompanied by Increased Seroreactivity. Retrovirology (2009) 6:104. doi: 10.1186/1742-4690-6-104 19917105 PMC2780989

[22] Sotgiu S Arru G Mameli G Serra C Pugliatti M Rosati G. Multiple Sclerosis-Associated Retrovirus in Early Multiple Sclerosis: A Six-Year Follow-Up of a Sardinian Cohort. Mult Scler Houndmills Basingstoke Engl (2006) 12:698–703. doi: 10.1177/1352458506070773 17262996

[23] Sotgiu S Mameli G Serra C Zarbo IR Arru G Dolei A. Multiple Sclerosis-Associated Retrovirus and Progressive Disability of Multiple Sclerosis. Mult Scler Houndmills Basingstoke Engl (2010) 16:1248–51. doi: 10.1177/1352458510376956 20685761

[24] Hartung H-P Derfuss T Cree BA Sormani MP Selmaj K Stutters J. Efficacy and Safety of Temelimab in Multiple Sclerosis: Results of a Randomized Phase 2b and Extension Study. Mult Scler Houndmills Basingstoke Engl (2021) 13524585211024996. doi: 10.1177/13524585211024997 34240656

[25] Malhotra S Castilló J Bustamante M Vidal-Jordana A Castro Z Montalban X. SIGLEC1 and SIGLEC7 Expression in Circulating Monocytes of Patients With Multiple Sclerosis. Mult Scler J (2013) 19:524–31. doi: 10.1177/1352458512458718 22933622

[26] Giménez-Orenga K Oltra E. Human Endogenous Retrovirus as Therapeutic Targets in Neurologic Disease. Pharm Basel Switz (2021) 14. doi: 10.3390/ph14060495 PMC8225122 34073730

[27] Charvet B Pierquin J Brunel J Gorter R Quétard C Horvat B. Human Endogenous Retrovirus Type W Envelope From Multiple Sclerosis Demyelinating Lesions Shows Unique Solubility and Antigenic Characteristics. Virol Sin (2021) 36(5):1006–26. doi: 10.1007/s12250-021-00372-0 PMC8558138 33770381

[28] Antony JM Ellestad KK Hammond R Imaizumi K Mallet F Warren KG. The Human Endogenous Retrovirus Envelope Glycoprotein, Syncytin-1, Regulates Neuroinflammation and its Receptor Expression in Multiple Sclerosis: A Role for Endoplasmic Reticulum Chaperones in Astrocytes. J Immunol Baltim Md 1950 (2007) 179:1210–24. doi: 10.4049/jimmunol.179.2.1210 17617614

[29] Duperray A Barbe D Raguenez G Weksler BB Romero IA Couraud P-O. Inflammatory Response of Endothelial Cells to a Human Endogenous Retrovirus Associated With Multiple Sclerosis is Mediated by TLR4. Int Immunol (2015) 27:545–53. doi: 10.1093/intimm/dxv025 PMC4625887 25957268

[30] Reynolds JM Martinez GJ Chung Y Dong C. Toll-Like Receptor 4 Signaling in T Cells Promotes Autoimmune Inflammation. Proc Natl Acad Sci USA (2012) 109:13064. doi: 10.1073/pnas.1120585109 22826216 PMC3420161

[31] Ramasamy R Joseph B Whittall T. Potential Molecular Mimicry Between the Human Endogenous Retrovirus W Family Envelope Proteins and Myelin Proteins in Multiple Sclerosis. Immunol Lett (2017) 183:79–85. doi: 10.1016/j.imlet.2017.02.003 28189601

[32] Ramasamy R Mohammed F Meier U-C. HLA DR2b-Binding Peptides From Human Endogenous Retrovirus Envelope, Epstein-Barr Virus and Brain Proteins in the Context of Molecular Mimicry in Multiple Sclerosis. Immunol Lett (2019) 217:15–24. doi: 10.1016/j.imlet.2019.10.017 31689443

[33] de Luca V Martins Higa A Malta Romano C Pimenta Mambrini G Peroni LA Trivinho-Strixino F. Cross-Reactivity Between Myelin Oligodendrocyte Glycoprotein and Human Endogenous Retrovirus W Protein: Nanotechnological Evidence for the Potential Trigger of Multiple Sclerosis. Micron Oxf Engl 1993 (2019) 120:66–73. doi: 10.1016/j.micron.2019.02.005 30802755

[34] Laufer G Mayer J Mueller BF Mueller-Lantzsch N Ruprecht K. Analysis of Transcribed Human Endogenous Retrovirus W Env Loci Clarifies the Origin of Multiple Sclerosis-Associated Retrovirus Env Sequences. Retrovirology (2009) 6:37. doi: 10.1186/1742-4690-6-37 19368703 PMC2672075

[35] Schmitt K Richter C Backes C Meese E Ruprecht K Mayer J. Comprehensive Analysis of Human Endogenous Retrovirus Group HERV-W Locus Transcription in Multiple Sclerosis Brain Lesions by High-Throughput Amplicon Sequencing. J Virol (2013) 87:13837–52. doi: 10.1128/JVI.02388-13 PMC3838257 24109235

[36] Elkjaer ML Frisch T Tonazzolli A Röttger R Reynolds R Baumbach J. Unbiased Examination of Genome-Wide Human Endogenous Retrovirus Transcripts in MS Brain Lesions. Mult Scler Houndmills Basingstoke Engl (2021) 27(12):1829–37. doi: 10.1177/1352458520987269 33464158

[37] Rizzo R Di Luca D. Human Herpesvirus 6A and 6B and NK Cells. Acta Microbiol Immunol Hung (2018) 65:119–25. doi: 10.1556/030.65.2018.010 29512392

[38] Eliassen E Di Luca D Rizzo R Barao I. The Interplay Between Natural Killer Cells and Human Herpesvirus-6. Viruses (2017) 9:E367. doi: 10.3390/v9120367 29194419 PMC5744142

[39] Charvet B Reynaud JM Gourru-Lesimple G Perron H Marche PN Horvat B. Induction of Proinflammatory Multiple Sclerosis-Associated Retrovirus Envelope Protein by Human Herpesvirus-6A and CD46 Receptor Engagement. Front Immunol (2018) 9:2803. doi: 10.3389/fimmu.2018.02803 30574140 PMC6291489

[40] Mameli G Astone V Arru G Marconi S Lovato L Serra C. Brains and Peripheral Blood Mononuclear Cells of Multiple Sclerosis (MS) Patients Hyperexpress MS-Associated Retrovirus/HERV-W Endogenous Retrovirus, But Not Human Herpesvirus 6. J Gen Virol (2007) 88:264–74. doi: 10.1099/vir.0.81890-0 17170460

[41] Römer C. Viruses and Endogenous Retroviruses as Roots for Neuroinflammation and Neurodegenerative Diseases. Front Neurosci (2021) 15:648629. doi: 10.3389/fnins.2021.648629 33776642 PMC7994506

[42] Geginat J Paroni M Pagani M Galimberti D De Francesco R Scarpini E. The Enigmatic Role of Viruses in Multiple Sclerosis: Molecular Mimicry or Disturbed Immune Surveillance? Trends Immunol (2017) 38:498–512. doi: 10.1016/j.it.2017.04.006 28549714 PMC7185415

[43] Bergallo M Galliano I Montanari P Zaniol E Graziano E Calvi C. Modulation of Human Endogenous Retroviruses -H, -W and -K Transcription by Microbes. Microbes Infect (2020) 22(8):366–70. doi: 10.1016/j.micinf.2020.01.006 32035224

[44] Song Y Li X Wei X Cui J. Human Endogenous Retroviruses as Biomedicine Markers. Virol Sin (2021) 36(5):852–8. doi: 10.1007/s12250-021-00387-7 PMC8558139 33905075

[45] Lezhnyova VR Martynova EV Khaiboullin TI Urbanowicz RA Khaiboullina SF Rizvanov AA. The Relationship of the Mechanisms of the Pathogenesis of Multiple Sclerosis and the Expression of Endogenous Retroviruses. Biology (2020) 9. doi: 10.3390/biology9120464 PMC7764762 33322628

[46] Domínguez-Mozo MI Nieto-Guerrero A Pérez-Pérez S García-Martínez MÁ Arroyo R Álvarez-Lafuente R. MicroRNAs of Human Herpesvirus 6A and 6B in Serum and Cerebrospinal Fluid of Multiple Sclerosis Patients. Front Immunol (2020) 11:2142. doi: 10.3389/fimmu.2020.02142 33072077 PMC7531184

[47] Arru G Caggiu E Leoni S Mameli G Pugliatti M Sechi GP. Natalizumab Modulates the Humoral Response Against HERV-Wenv73-88 in a Follow-Up Study of Multiple Sclerosis Patients. J Neurol Sci (2015) 357:106–8. doi: 10.1016/j.jns.2015.07.007 26190523

[48] Mameli G Cossu D Cocco E Frau J Marrosu MG Niegowska M. Epitopes of HERV-Wenv Induce Antigen-Specific Humoral Immunity in Multiple Sclerosis Patients. J Neuroimmunol (2015) 280:66–8. doi: 10.1016/j.jneuroim.2015.03.003 25773158

[49] Engdahl E Gustafsson R Huang J Biström M Lima Bomfim I Stridh P. Increased Serological Response Against Human Herpesvirus 6a Is Associated With Risk for Multiple Sclerosis. Front Immunol (2019) 10:2715. doi: 10.3389/fimmu.2019.02715 32038605 PMC6988796

