# Supplementary material for: Anti-Human Herpesvirus 6 A/B Antibodies Titers Correlate With Multiple Sclerosis-Associated Retrovirus Envelope Expression
Source: Front Immunol. 2021 Nov 29;12:798003. doi: 10.3389/fimmu.2021.798003 (PMC8666430; doi:10.3389/fimmu.2021.798003)
Supplement: Supplementary file 1 [file DataSheet_1.pdf]

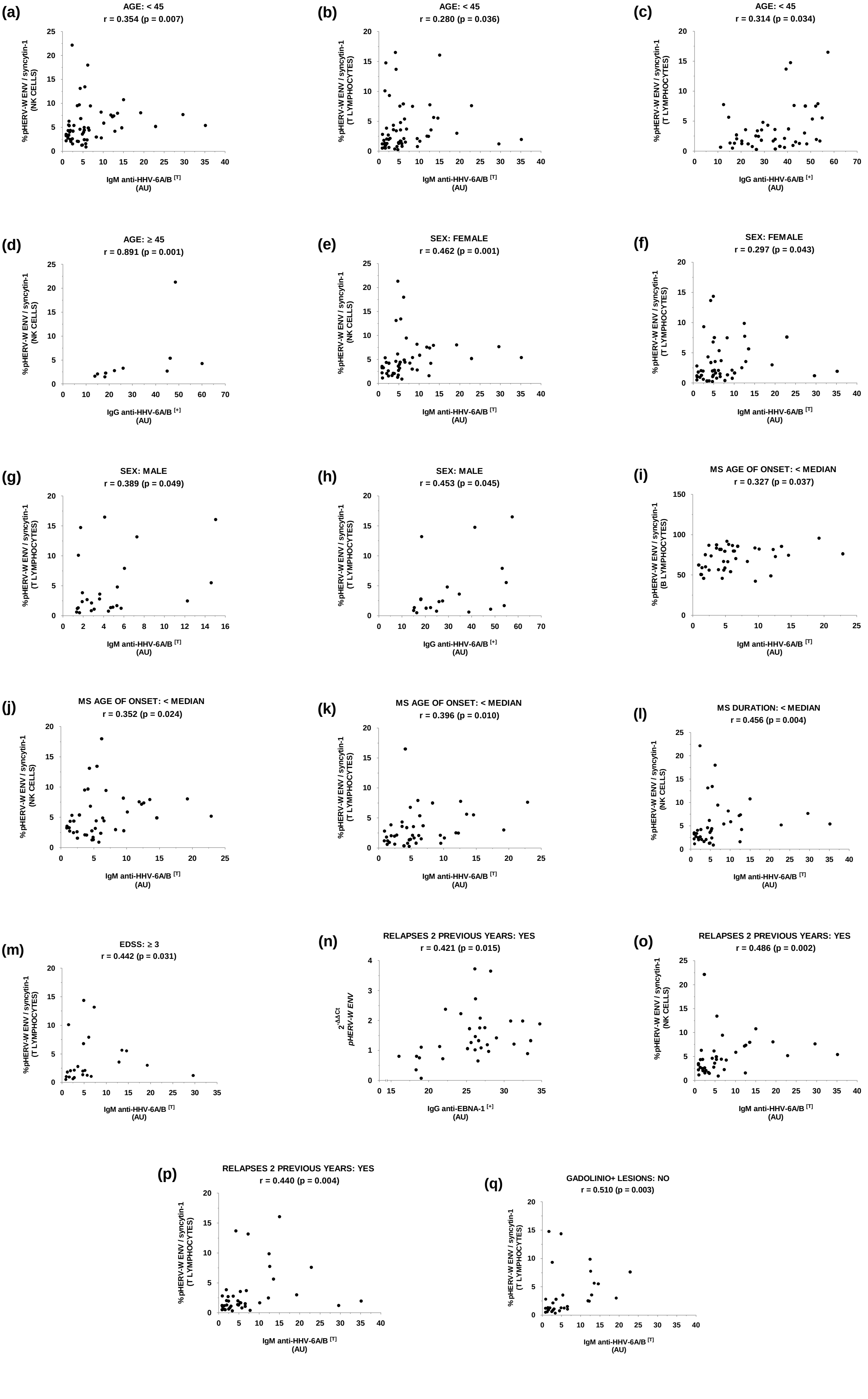

Supplementary Figure 1. Influences of demo-graphical, clinical, and radiological factors on correlations between antiviral antibodies titers and pHERV-W ENV/syncytin-1 expression.
